# 38.1 IMPACT OF ANTI-NMDA RECEPTOR AUTOANTIBODIES FROM PSYCHOTIC PATIENTS ON THE GLUTAMATE SYNAPSE

**DOI:** 10.1093/schbul/sby014.154

**Published:** 2018-04-01

**Authors:** Laurent Groc

**Affiliations:** CNRS, University of Bordeaux

## Abstract

**Background:**

The flourishing identification of circulating autoantibodies against neuronal receptors in neuropsychiatric disorders has fostered new conceptual and clinical frameworks. However, their putative presence in different diseases, as well as in healthy subjects, has raised questions about detection reliability and pathogenic role.

**Methods:**

Using a combination of single molecule-based imaging approaches, cell calcium imaging, and single-cell electrophysiological recordings, we investigated in hippocampal networks the impact of autoantibodies against glutamate NMDA receptor (NMDAR-Ab) on several aspects of the glutamate synapse.

**Results:**

We ascertain the presence of circulating autoantibodies against glutamate NMDA receptor (NMDAR-Ab) in about 20% of psychotic patients diagnosed with schizophrenia and very few healthy subjects. NMDAR-Ab from patients and healthy subjects do not compete for binding on native receptor. Strikingly, NMDAR-Ab from patients, but not from healthy subjects, specifically alter the surface dynamics and nanoscale organization of synaptic NMDAR and its anchoring partner the EphrinB2 receptor. Functionally, only patients’ NMDAR-Ab prevent long-term potentiation at glutamatergic synapses while leaving NMDAR-mediated calcium influx intact. Furthermore, we unveil that NMDAR-Ab from first episode psychotic patients produced similar effects.

**Discussion:**

By taking advantage of the single molecule imaging and complementary ensemble approaches, we unveil that NMDAR-Ab from psychotic patients (schizophrenic and first episode) profoundly alter NMDAR synaptic transmission and NMDAR-dependent synaptic functions, supporting a pathogenically relevant role.

